# Thoracoscopic capitonnage for pulmonary hydatid cysts: the predictors of prolonged air leak

**DOI:** 10.3389/fsurg.2025.1664976

**Published:** 2025-10-28

**Authors:** Fahmi H. Kakamad

**Affiliations:** 1 Department of Cardiothoracic and Vascular Surgery, College of Medicine, University of Sulaimani, Sulaymaniyah, Iraq; 2 Scientific Affairs Department, Smart Health Tower, Sulaymaniyah, Iraq; 3 Kscien Organization for Scientific Research (Middle East Office), Sulaymaniyah, Iraq

**Keywords:** hydatid disease, echinococcosis, VATS, cystotomy, capitonnage, atelectasis

## Abstract

**Objectives:**

Pulmonary hydatid cysts (PHCs) represent a serious zoonotic disease that requires prompt intervention because of their potential complications. Capitonnage is one of the most common performed techniques; however, controversies surround its role in managing PHCs. The present study aims to evaluate lung parenchyma using computed tomography (CT) scans 48 h after capitonnage of PHCs performed via VATS.

**Methods:**

Fifty-six patients with suspected PHCs on CT scans between 2021 and 2023 were included, while we excluded patients with risk factors for prolonged air leak (PAL), patients with other organ involvement, those presenting with emergency conditions, and those younger than 12 years old.

**Results:**

The CT scans revealed residual cavities in all 56 patients (100%), ranging in size from 2 to 12 cm. There was a collapse consolidation involving the affected lobe in all of the cases (100%). Eight patients (14%) experienced PAL; one patient (12.5%) had an intact cyst, while the other seven cases (87.5%) had ruptured cysts (*p*-value <0.001). There was no significant difference in cavity size between patients who developed PAL and those without PAL (*p*-value: 0.07), while patients with larger areas of consolidation tended to have PAL (*p*-value: 0.001).

**Conclusions:**

The PAL is more likely caused by collapse consolidation rather than residual cavities left after the procedure. Additionally, ruptured cysts can significantly contribute to the complication of PAL.

## Graphical Abstract

**Figure:**
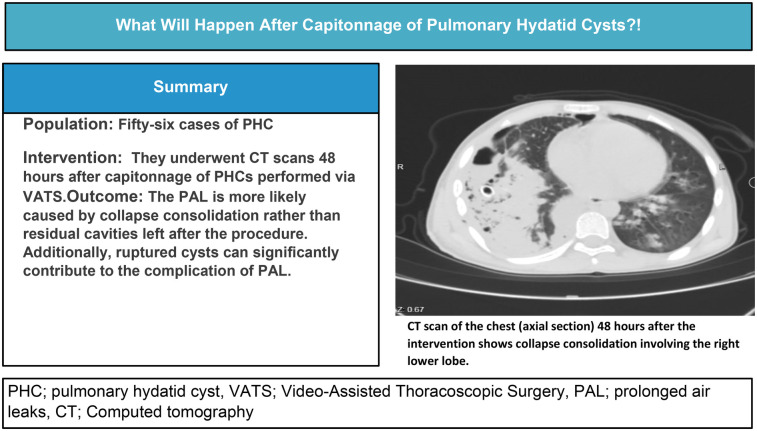

## Introduction

Pulmonary hydatid cysts (PHCs), caused by the larval stage of *Echinococcus* tapeworms, remain a significant zoonotic disease, primarily in regions where animal husbandry practices are prevalent and preventive measures are inadequate (1, 2). Nine species of *Echinococcus* have been identified, among which four are of significant public health concern: *Echinococcus granulosus*, the causative agent of cystic echinococcosis; *Echinococcus multilocularis*, responsible for alveolar echinococcosis; and *Echinococcus vogeli* and *Echinococcus oligarthrus*, both associated with polycystic echinococcosis (3, 4). There are varying incidence rates of the disease across different geographical regions. Australia, New Zealand, South America, Turkey, and Mediterranean countries report the highest incidence rates due to their agricultural and livestock-rearing practices (5–7). The livestock farming and agricultural practices in Iraq are factors that also increase the risk of disease spreading among the population, which is also exacerbated by the lack of effective control measures (8).

Cystic echinococcosis requires both definitive and intermediate hosts. Canids and dogs are the definitive hosts, harboring the adult tapeworm and shedding their eggs in the feces. The grazing hosts, including sheep, goats, and cattle, ingest the eggs that release the oncospheres and migrate to the lungs and liver, and develop hydatid cysts. Humans are accidental intermediate hosts and become infected through the ingestion of eggs through contaminated water or food or through physical contact with dogs (9). Although almost all organs could be affected, the liver and lungs are the most commonly affected organs, with the lung being the second most frequent site of infection (10, 11). The HCs often remain asymptomatic for extended periods and may be incidentally discovered during imaging studies or present with symptoms depending on the cyst's size, location, and complications (12). Common clinical manifestations include cough, chest pain, and respiratory distress, while complications such as cyst rupture can lead to anaphylaxis or disseminating daughter cysts to other organs (5). Once diagnosed by imaging or serology, surgical intervention remains the cornerstone of treatment (10). Various surgical approaches have been described, including cystotomy, capitonnage, pericystectomy, and segmentectomy, tailored to the cyst's characteristics and location within the lung (1). However, capitonnage is one of the most performed techniques; controversies surround its role in PHC management. Capitonnage has been advocated to prevent complications such as prolonged air leaks (PAL) and empyema formation (1). However, others suggest that simple closure of bronchial openings without capitonnage may be enough to avoid potential lung parenchymal disfigurement and atelectasis (13). The debate continues regarding the necessity and effectiveness of capitonnage in managing PHCs (14). Advancements in minimally invasive techniques, particularly video-assisted thoracoscopic surgery (VATS), have revolutionized the thoracic and pulmonary surgery in general (15). It offers advantages over traditional open surgery, including reduced postoperative pain, shorter hospital stays, and comparable efficacy in cyst removal while preserving lung function (16). However, the use of VATS for managing PHCs is still controversial due to factors such as the size of the cyst cavity, severe adhesions, hemoptysis, PAL, and challenges in controlling bronchopleural fistulas (13, 14).

The present study aims to evaluate lung parenchyma using a computed tomography (CT) scan 48 h after capitonnage of PHCs performed via VATS.

## Patients and methods

### Ethical statement

The study has been approved by the ethics committee of the College of Medicine, University of Sulaimani. Written informed consent was obtained from all patients to participate in and publish their data in the study.

### Study design and setting

This single-group cohort study enrolled consecutive patients diagnosed provisionally with PHCs during two years (2021–2023) of practice in a single tertiary center.

### Eligibility criteria

**Figure 1:**
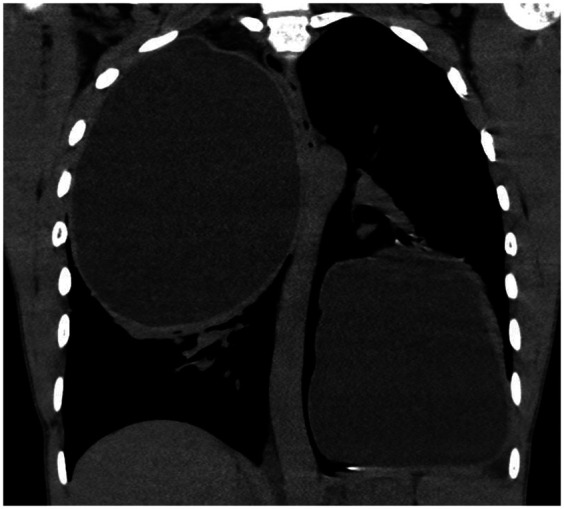
Computed tomography (CT) scan of the chest (coronal section) showing bilateral large cysts with homogenous fluid density and peripheral rim enhancement, suggesting bilateral giant hydatid cysts.

All patients with suspected PHCs on CT scan were included (Figure 1). Patients with risk factors for PAL [such as chronic obstructive pulmonary disease, steroid use, or forced expiratory volume in one second (FEV1) lower than 80%], patients with other organ involvement, those presenting with emergency conditions (such as pneumothorax), and those younger than 12 years old (due to difficulty in performing a CT scan) were excluded.

### Surgical intervention

**Figure 2:**
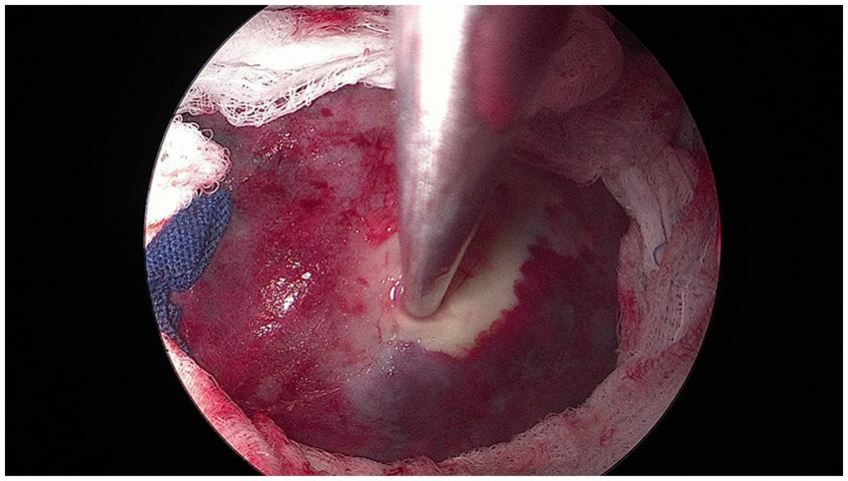
Intraoperative photo showing a large hydatid cyst isolated with packs soaked with normal saline.

According to hospital protocol, patients were given a single dose of ciprofloxacin (400 mg infusion) one hour before induction. Under general anesthesia, the patients underwent bi-port VATS while positioned laterally with double-lumen endotracheal intubation. The camera incision was made in the seventh intercostal space along the anterior axillary line. This incision was later used for inserting the chest tube. The utility incision was created in the ipsilateral thorax, taking into account the number and location of the HCs. The cyst was utterly isolated and evacuated using packs soaked in hypertonic saline, followed by washing the cyst and cavity with hypertonic saline (Figure 2). Vicryl 2.0 was used to close the fistulae. Capitonnage (purse-string sutures) was done for all of the patients, with the extent standardized according to the cavity size. A single 28-French chest drain was inserted at the end of the procedure. If a patient had bilateral HCs, he/she was repositioned, and the same procedure was repeated for the other side. All patients were extubated in the operating room.

### Follow-up

**Figure 3:**
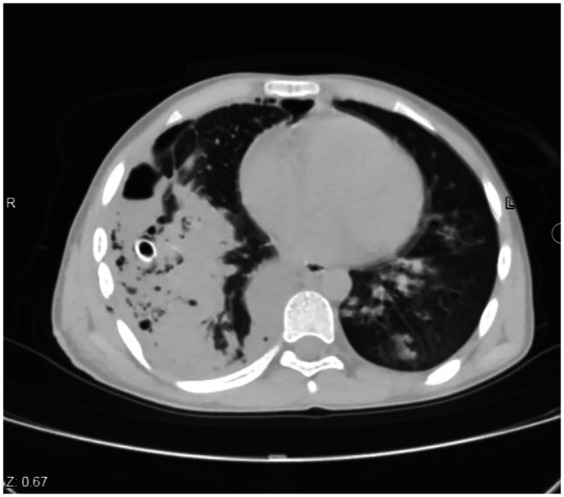
Computed tomography (CT) scan of the chest (axial section) 48 h after the intervention shows collapse consolidation involving the right lower lobe.

Oral intake and early mobilization started after the patient's complete recovery in the ward (3–6 h after extubation). The patients were administered ciprofloxacin 400 mg × 2 intravenously (IV), acetaminophen 1 gm × 4 IV, ketorolac ampule 30 mg × 3, and pethidine as required. They also received albendazole tablets 400 mg × 2. Forty-eight hours after the procedure, a chest CT scan was requested and examined (Figure 3). PAL was defined as the persistence of air leakage from the residual cyst cavity into the pleural space for more than 7 days after surgery, despite appropriate chest tube drainage (17). Postoperatively, complete blood count and C-reactive protein were conducted for all the cases to exclude anemia, inflammation, or infection. Both laboratory tests were in normal ranges.

### Data analysis

The data was extracted from the hospital database and transferred to a Microsoft Excel spreadsheet (2013 version). The data were presented as mean, range, frequency and percentages. Categorical data were compared using the Chi-square test, while numerical data were analyzed using the *T*-test. The significance level was set at less than 0.05.

## Results

The total number of patients was 56. Of these, 32 patients (57%) were male, and the age range was 12–60 years, with a mean age of 35. Thirty-one patients (55%) had a history of pet/animal contact. Thirty-seven patients (66%) were asymptomatic. The right side was involved in 34 (61%) patients, the left in 15 (27%) patients, and bilateral in the remaining cases (7 cases, 12%). The most frequently affected site was the right lower lobe (14 cases, 25%). The number of cysts ranged from 1 to 12, with a mean of 3. The size of the cysts ranged from 3 to 16 cm, with an average of 7 cm. The CT scans revealed residual cavities in all patients, ranging in size from 2 to 12 cm, with a mean of 5 cm. There was a collapse consolidation involving the affected lobe in all of the cases. The sizes ranged from 1 to 5 cm, with an average of 3 cm (Table 1). The cysts were intact in 39 patients (70%) and ruptured in the remaining cases (30%). Eight patients (14%) experienced PAL; one patient (12.5%) had an intact cyst, while the other seven cases (87.5%) had ruptured cysts (*p*-value <0.001). There was no significant difference in cavity size between patients who developed PAL and those without PAL (*p*-value: 0.07). In contrast, patients with larger areas of consolidation tended to have PAL (*p*-value: 0.001).

**Table 1:** The baseline characteristics and clinical data of the participants.

Variables	Frequency/Percentage
Patient demographics	
Age range (mean), years	12–60 (35)
Gender	
Male	32 (57.0)
Female	24 (43.0)
History of contact with pets	
Yes	31 (55.0)
No	25 (45.0)
Symptoms	
Asymptomatic (accidental findings)	37 (66.0)
Signs and symptoms of chest infection	19 (34)
The involved side	
Right	34 (61.0)
Left	15 (27.0)
Bilateral	7 (12.0)
The involved site	
Right lower lobe	14 (25.0)
More than one lobe	13 (23.0)
Left lower lobe	10 (18.0)
Right upper lobe	9 (16.0)
Left upper lobe	8 (14.0)
Middle lobe	2 (4.0)
Number of the cysts, range (mean)	1–12 (3)
Size of the cyst (cm), range (mean)	3–16 (7)
Cavity and collapse consolidation	
Yes	56 (100)
No	0 (0.0)
Size of cavities (cm), range (mean)	2–12 (5)
Size of collapse consolidation (cm), range (mean)	1–5 (3)
Cyst status	
Intact cyst	39 (70.0)
Ruptured cyst	17 (30.0)
Prolonged air leaks	
Yes	8 (14.0)
No	48 (86.0)
Hospital stays range (mean)	2.5 (2–13)
Recurrence	0 (0)

## Discussion

Preventing HCs requires a comprehensive strategy. Control of the disease primarily relies on preventing the transmission of *E. granulosus* eggs from dogs or contaminated food and water to humans, and on avoiding the ingestion of hydatid cysts in inedible offal by dogs and other canines. This includes deworming livestock, properly disposing of infected animal carcasses, encouraging good hygiene practices, especially among livestock handlers, properly cooking meat, and raising community education (18).

Hydatid disease can infect individuals of any age or sex, but it is most commonly observed in people between the ages of 20 and 40. Individuals within this age range are often more actively involved in livestock rearing and agricultural practices, which increases their risk of exposure to the parasite egg (8). In a study by Turna et al., involving 75 patients, the average age was found to be 30.2 ± 17.4 years (19). The predominance of the disease in males has been well-documented. This trend may be attributed to males' earlier and more frequent contact with dogs (5). Ahmed et al. found a predominance of males (61.9%) over females (38.1%) in their study. In contrast, Goni et al. reported opposite findings, with a 65% female and 35% male distribution (5, 20). In the present study, consistent with the literature, the mean age of the patients was 35 years, and males constituted 57% of the sample size.

The PHCs can potentially develop in any lobe of the lungs, yet the right and lower lobes are frequently affected (5). Approximately 60% of cases occur in the lower lobes of the right lung (5). In the study by Alpay et al., all four patients presented with involvement of the lower lobes (21). It has been documented that 8% to 32% of patients with hydatid disease are asymptomatic. According to Yaldiz et al., the proportion of asymptomatic patients was 22.4% across all cases, with cough and chest pain being the most frequently reported symptoms (17). In the study by Aldahmashi et al., which involved 148 cases of PHCs, the presenting symptoms varied widely, ranging from asymptomatic to severe symptoms such as massive hemoptysis. Approximately 10.8% of patients were asymptomatic, and the most common presenting symptom was cough (73.6%), followed by dyspnea (62.2%), chest pain (64.9%), and fever (14.9%) (12). In this study, the most commonly involved site was the right lower lobe (25%), followed by multilobe involvement (23%) and the left lower lobe (18%). In total, 66% of the cases were asymptomatic, and 34% presented with intermittent cough and chest pain.

Clinical assessments, chest radiography, CT scans, and *Echinococcus* serologic studies collectively aid in confirming the accurate preoperative diagnosis of PHCs. On chest x-rays, an uncomplicated HC is characterized by a well-defined, homogeneous radio-opacity, while a perforated cyst may display a distinctive “water lily” or “signet ring” sign. CT scans can identify PHCs by showing features such as the “inverted crescent sign”, “signet ring appearance”, high CT density, and thickened cyst wall (5, 22). In the current study, all cases were suspected of having PHCs based on the characteristics observed on CT scans, as described previously.

Various surgical techniques have been employed to excise HCs, repair lung tissue, and minimize complications and recurrence risks. Depending on the lesion's location and accessibility, procedures range from thoracotomy incisions of different sizes to VATS and hybrid interventions, though open surgery remains more common. The primary objectives of these surgical treatments are to remove the endocyst along with the daughter vesicles, preserve lung tissue, prevent cyst rupture at the surgical site, and close any bronchial openings in the cyst wall. Resection is not suggested unless the entire lobe is destroyed (1, 21). The Ugon enucleation technique allows cyst removal with its intact germinative membrane. This method is suitable for small cysts with a low risk of rupture, but it is not recommended for giant cysts due to the potential for postoperative air leaks and infections in the residual space (10, 23). Pericystectomy, another technique for HC surgery, has already been abandoned due to the risk of air leaks. The Barret method has made significant advances in HC surgery by introducing capitonnage, which involves obliterating the pericyst cavity after removing the cyst membrane. The Posadas method, a variation of Barrett's procedure, includes closing the bronchial openings before performing capitonnage. In this technique, the bronchial openings in the pericyst wall are closed with sutures. Then, the cyst cavity walls are obliterated using interrupted purse-string sutures for capitonnage. Eventually, with continuous sutures, the healthy parenchymal ends are approximated (10, 19). Since then, enucleation with capitonnage has been a controversial treatment for PHCs (12). The central debate in PHC surgery concerns whether capitonnage is necessary following cystotomy (13). Capitonnage is typically recommended for ensuring complete obliteration of residual space (14). However, particularly in the 2000s, studies have suggested that capitonnage may not be essential, advocating for the closure of bronchial leaks to allow the expansible parenchyma to heal (13, 19). Advocates of the capitonnage procedure emphasize its role in preventing postoperative air leaks and the formation of empyema. Conversely, opponents argue that it causes distorting pulmonary parenchyma, particularly after removing multiple cysts and closing the mouths of major bronchi, which may lead to atelectasis due to restricted lung re-expansion post-surgery (23). However, some studies counter this claim, reporting either no occurrence or a very low incidence of atelectasis following the capitonnage procedure (17, 24).

Despite the significant advancements in thoracoscopic surgery, the majority of surgeons continue to prefer the traditional open thoracotomy approach for treating PHCs. This preference may stem from concerns about controlling the cyst's contents during surgery, which could potentially rupture into the pleural cavity, leading to contamination or anaphylactic reactions. However, with increasing experience in VATS, it has been demonstrated that this approach can also be safely utilized for non-perforated or live HCs with lower postoperative pain, shorter hospital stays, and complications (15, 21). In a case series study of four cases undergoing VATS removal of PHCs with capitonnage, no complications were reported during the procedures and in a 4-month follow-up period (21). Ma et al. conducted a comparison between thoracoscopic and open-thoracic pulmonary hydatid cystotomy. Their findings indicated that the thoracoscopic approach offers several benefits, including reduced trauma, decreased bleeding, lower postoperative drainage flow, diminished pain, a lower incidence of complications, and reduced hospitalization costs (25).

Ksia et al. assessed thoracotomy outcomes in pediatric cases with and without capitonnage. They observed pneumothorax and emphysema in 30% of cases without capitonnage compared to 13.2% in cases with capitonnage. Residual cavity persistence was found in 23.3% of cases without capitonnage and 7.9% in cases with capitonnage (14). A meta-analysis comparing the outcomes of capitonnage and uncapitonnage techniques for PHCs found that complications were significantly less frequent in the capitonnage group compared to the uncapitonnage group, with a relative risk reduction of 3.81 times. Specifically, the incidence of PAL and empyema was reduced by 4.18 and 4.76 times, respectively, in favor of the capitonnage technique. However, there was no significant difference between the two groups regarding the occurrence of atelectasis or the length of hospital stay (1). Amine et al. also concluded that capitonnage significantly reduces the incidence of pneumothorax, emphysema formation, and residual cavities compared to non-capitonnage techniques in the long term (26). Yaldiz et al. reported PAL in 10 cases who underwent thoracotomy; seven (70%) were in the uncapitonnage group (17). Conversely, some scholars have reported that non-capitonnage approaches resulted in shorter hospital stays, less drainage time, better radiologic improvement, and fewer cases of pleural empyema and morbidity (13). In the present study, 56 cases of PHCs were treated using bi-port VATS with capitonnage. To the best of our knowledge, this represents one of the largest series of purely thoracoscopic repair of PHCs with capitonnage, following studies by Zairi et al. and Abbas et al., which included 511 and 120 cases, respectively, but their techniques were associated with mini-thoracotomy (27, 28). The average number of cysts was 3 cm, with an average size of 7 cm. Residual cavity and collapse consolidation were present in all 56 cases, with average sizes of 5 cm and 3 cm, respectively. Most of the cysts (70%) were intact. The PAL occurred in eight cases (14%), one with an intact cyst and seven with ruptured cysts, which reached a significant difference. The incidence of PAL (14%) was higher than that reported in several published PHCs and VATS series, which typically report PAL rates of 1%–4% following parenchyma-preserving techniques with routine capitonnage. Incidence rates between 5% and ≥10% have also been observed in series that include complicated or ruptured cysts, pediatric patients, or open thoracotomy cases (1, 17, 28). Variations in PAL definitions (postoperative day >5 vs. >7), case mix (giant, ruptured, or infected cysts), surgical technique (capitonnage vs. uncapitonnage; meticulous closure of bronchial openings), operative approach (VATS experience, uniportal vs. multiport), and postoperative drainage protocols likely explain these differences (17, 28).

Our findings revealed that it was not the residual cavities that caused PAL, as traditionally believed, but rather the collapse consolidation that significantly contributed to that complication. However, the potential impact of the small sample size cannot be ruled out. With larger cohorts, the findings may differ. The mean hospital stay was 2.5 days.

Postoperative collapse consolidation (atelectasis) is a recognized contributor to PAL after PHC surgery. It results from reduced lung compliance, impaired cough and deep breathing, increased risk of infection, and potential formation of a bronchial fistula, all of which sustain pleural air leakage. Preventive strategies include early physiotherapy with deep breathing and forced coughing, which decrease atelectasis and improve pulmonary function (29). Additionally, early mobilization has been shown to enhance lung expansion, reduce pain, and improve respiratory outcomes, although with variable effects on PAL rates (30). Adequate analgesia, using multimodal or regional techniques, facilitates effective coughing and breathing (31). In addition, close monitoring for early atelectasis and timely interventions such as bronchoscopy or chest tube adjustment can prevent progression to PAL (31). Standardizing these measures in postoperative care protocols may significantly reduce collapse consolidation and associated PAL after capitonnage.

In a study by Erdogan et al., three recurrences were observed in the capitonnage group and five recurrences in the non-capitonnage group (32). Kosar et al. reported recurrence rates of 6.5% in children treated with capitonnage and 5.6% in those treated with cystotomy alone during the follow-up period, with no statistically significant difference between the groups (24). In another study, the recurrence rate was 0% in the cystotomy with capitonnage group compared to 1.7% in the cystotomy alone group (14). In the present study, no cases of recurrence were reported. The present study is constrained by several limitations that reduce its generalizability, including a limited sample size that prevents robust statistical validation, the inclusion criteria (the study population might be healthier than the general population of patients with PHCs), the absence of a control group, and the lack of collecting preoperative assessment data and long-term follow-up data.

## Conclusion

After capitonnage of PHC via VATS, PAL is more likely caused by collapse consolidation rather than residual cavities left after the procedure. Additionally, ruptured cysts can significantly contribute to the complication of PAL. Further studies may be necessary to shed further light on the subject.

## Data Availability

The original contributions presented in the study are included in the article/Supplementary Material, further inquiries can be directed to the corresponding author.
